# Pathway-Specific Therapeutic Modulation of Melanoma: Small-Molecule Inhibition of BRAF–MEK and KIT Signaling in Contemporary Precision Oncology with a Special Focus on Vemurafenib, Trametinib, and Imatinib

**DOI:** 10.3390/jcm14227906

**Published:** 2025-11-07

**Authors:** Piotr Kawczak, Tomasz Bączek

**Affiliations:** 1 Department of Pharmaceutical Chemistry, Faculty of Pharmacy, Medical University of Gdańsk, 80-416 Gdańsk, Poland; tomasz.baczek@gumed.edu.pl; 2 Department of Nursing and Medical Rescue, Institute of Health Sciences, Pomeranian University in Słupsk, 76-200 Słupsk, Poland

**Keywords:** melanoma, targeted treatment, vemurafenib, trametinib, imatinib, BRAFi, MEKi, C-KITi

## Abstract

Melanoma is an aggressive form of skin cancer marked by unique genetic alterations that promote tumor growth and resistance to therapy. Advances in targeted therapy have markedly improved clinical outcomes by selectively inhibiting key oncogenic pathways. This review focuses on three clinically relevant agents—vemurafenib, trametinib, and imatinib—analyzing their mechanisms of action, clinical applications, efficacy, and limitations. Vemurafenib, a selective BRAFV600E inhibitor, significantly extends progression-free and overall survival in BRAF-mutant melanoma but is limited by acquired resistance and frequent cutaneous toxicities. Trametinib, a MEK1/2 inhibitor, acts downstream in the MAPK pathway and is typically combined with BRAF inhibitors to enhance efficacy and delay resistance. Imatinib, targeting c-KIT and PDGFR mutations, demonstrates therapeutic benefit primarily in acral and mucosal melanoma subtypes, though with lower response rates than BRAF-directed therapies. Adverse events associated with these drugs are generally manageable with appropriate monitoring. Despite substantial advances, secondary mutations and reactivation of oncogenic signaling remain major challenges. This narrative review integrates data from clinical, preclinical, and real-world studies to update the current understanding of targeted therapies in cutaneous melanoma and highlight ongoing research aimed at overcoming resistance and optimizing personalized treatment strategies.

## 1. Introduction

Cutaneous melanoma is a severe and rapidly progressing skin malignancy of melanocytic origin and represents the most lethal form of skin cancer. According to recent epidemiologic data, its global incidence continues to rise, with more than 320,000 new cases and approximately 57,000 deaths reported annually. Major risk factors include ultraviolet (UV) radiation exposure, fair skin phenotype, genetic predisposition, and the presence of dysplastic nevi. Despite significant therapeutic progress, advanced melanoma remains associated with high morbidity and mortality due to its strong metastatic potential and frequent development of therapeutic resistance [1,2,3].

The efficacy of approved small-molecule inhibitors in melanoma is primarily determined by the presence of actionable mutations—most notably BRAF V600 and activating KIT alterations—rather than by the tumor’s anatomical site alone. BRAF + MEK inhibitors demonstrate the greatest clinical benefit in BRAF V600–mutant cutaneous melanomas, achieving high response rates (~65–75%) and significant improvements in survival outcomes. Acral and mucosal melanomas, although less frequently harboring BRAF V600 mutations, can also respond to these inhibitors when such mutations are present. In contrast, KIT-mutant acral and mucosal melanomas derive modest but meaningful benefit from KIT inhibitors (e.g., imatinib, nilotinib), particularly when activating mutations occur in sensitive regions such as exon 11, though responses are typically less durable than those achieved with BRAF + MEK therapy. Uveal melanoma, characterized by GNAQ, GNA11, CYSLTR2, or PLCB4 mutations, remains largely refractory to both BRAF- and KIT-directed therapies and requires alternative, non-MAPK-targeted strategies. Overall, inhibitor performance in melanoma is driven by molecular genotype and influenced by subtype, with BRAF/MEK inhibitors offering the strongest benefit in BRAF V600–mutant cutaneous disease and KIT inhibitors providing a limited but targeted option for select non-cutaneous subtypes [4].

The molecular characterization of cutaneous melanoma has revealed key driver mutations that activate oncogenic signaling pathways, most notably the mitogen-activated protein kinase (MAPK) cascade. Approximately 40–60% of cutaneous melanomas harbor BRAF mutations—predominantly the V600E variant—which constitutively activates MAPK signaling, promoting tumor proliferation and survival [5,6,7,8,9,10,11]. This breakthrough enabled the development of small-molecule inhibitors that selectively target mutant BRAF, fundamentally reshaping the therapeutic landscape of melanoma. The advent of these agents marked a paradigm shift from nonspecific cytotoxic chemotherapy to mechanism-based, precision therapies guided by molecular profiling. Initially, clinical investigations centered on the selective inhibitors vemurafenib, trametinib, and imatinib; however, the therapeutic repertoire has since broadened considerably, with several additional small-molecule inhibitors now receiving regulatory approval for the treatment of melanoma.

Among these, the BRAF inhibitors—vemurafenib (Zelboraf), dabrafenib (Tafinlar), and encorafenib (Braftovi)—have shown remarkable efficacy in patients harboring BRAF V600E/K mutations, producing rapid tumor regression and high initial response rates [12,13,14]. However, these benefits are often short-lived, as acquired resistance commonly develops within months of treatment initiation. Preclinically, BRAF inhibitors (vemurafenib, dabrafenib, encorafenib) suppress ERK phosphorylation in BRAF V600-mutant models but differ in pharmacokinetics and paradoxical MAPK activation [15]. Clinically, dabrafenib and encorafenib have improved tolerability and lower cutaneous toxicity than vemurafenib [16].

Mechanisms of resistance include secondary mutations in NRAS or MEK1/2, BRAF amplification, alternative splicing, receptor tyrosine kinase (RTK) upregulation (e.g., epidermal growth factor receptor [EGFR] and platelet-derived growth factor receptor [PDGFR]), and compensatory activation of the phosphoinositide 3-kinase (PI3K)/AKT/mammalian target of the rapamycin (mTOR) pathway [17,18,19,20].

To counteract these resistance mechanisms, combination therapy with BRAF and MEK inhibitors has become the standard of care. Regimens such as dabrafenib plus trametinib and vemurafenib plus cobimetinib have demonstrated improvements in progression-free survival (PFS) and overall survival (OS), typically extending median PFS to 12–15 months while reducing paradoxical MAPK activation and adverse cutaneous events such as squamous cell carcinoma [21,22,23,24].

Although other signaling elements such as the KIT oncogene, the tumor microenvironment (TME), and immune checkpoint inhibitors (ICIs) play relevant roles in melanoma biology, their further discussion is presented in the subsequent section—“Cutaneous Melanoma Treatment and Mechanisms of Action”—to maintain focus in the introduction on the central role of BRAF-targeted therapy.

The introduction of small-molecule inhibitors has fundamentally shifted melanoma management from nonspecific cytotoxic chemotherapy toward mechanism-based, precision treatments guided by molecular profiling. Continued research is focused on understanding resistance mechanisms, optimizing combination regimens, and identifying predictive biomarkers to achieve more durable responses [25,26,27,28,29,30,31,32,33,34,35,36,37,38].

For the present synthesis, a narrative literature review was conducted using the PubMed and Scopus databases. The search strategy included the terms “vemurafenib,” “trametinib,” and “imatinib,” in combination with “targeted therapy” and “melanoma.” Peer-reviewed studies published between 2010 and 2025 were considered, with emphasis on clinical, translational, and mechanistic investigations. Articles were selected based on relevance, quality, and their contribution to understanding the therapeutic efficacy, mechanisms of action, and resistance patterns of these agents. Both preclinical and clinical studies were included when they provided mechanistic insights or informed therapeutic strategies. By applying these criteria, this review provides a balanced synthesis of current knowledge, highlighting both the clinical benefits and limitations of vemurafenib, trametinib, and imatinib in the management of cutaneous melanoma.

## 2. Cutaneous Melanoma Treatment and Mechanisms of Action

Targeted therapies have transformed the management of cutaneous melanoma by selectively inhibiting oncogenic signaling pathways. BRAF inhibitors, including vemurafenib, dabrafenib, and encorafenib, act by selectively binding the ATP-binding pocket of mutant BRAF (predominantly V600E). This suppresses constitutive MAPK pathway signaling, inhibits tumor proliferation, and induces apoptosis [11,12,13,14,15,16]. Clinical trials of vemurafenib demonstrated rapid tumor regression with overall response rates (ORR) of 50–60%, and improvements in progression-free survival (PFS) and overall survival (OS) in advanced BRAF-mutant melanoma [38,39,40]. Dabrafenib and encorafenib have shown similar efficacy, with encorafenib frequently combined with the MEK inhibitor binimetinib to enhance response and delay resistance [14,15,16].

The MEK inhibitors trametinib (Mekinist), cobimetinib (Cotellic), and binimetinib (Mektovi) thus play an essential role in combination strategies aimed at enhancing the durability of the response and mitigating toxicity. MEK inhibitors, such as trametinib and cobimetinib, act downstream of BRAF to inhibit MEK1/2, preventing ERK phosphorylation and cell proliferation [41,42,43]. While MEK inhibitor monotherapy provides modest benefits, combination therapy with BRAF inhibitors significantly improves PFS and OS. It also reduces paradoxical MAPK pathway activation, which lowers the incidence of BRAF inhibitor–associated cutaneous squamous cell carcinoma [44].

MEK inhibitors show modest single-agent activity but enhance responses when combined with BRAF inhibitors. Dabrafenib + trametinib, vemurafenib + cobimetinib, and encorafenib + binimetinib yield median overall survival >30 months. Real-world data support trial outcomes but are influenced by LDH levels, brain metastases, and performance status [15,16,45].

Despite these advances, resistance remains a major clinical challenge. Tumors can acquire secondary mutations in NRAS or MEK1/2, amplify BRAF, or undergo alternative splicing [46,47]. Upregulation of receptor tyrosine kinases (RTKs), including EGFR and PDGFR, further contributes to pathway reactivation [46,47,48]. Compensatory signaling through PI3K/AKT/mTOR also drives resistance [49,50,51]. In addition, non-genetic adaptations—such as tumor cell plasticity, epigenetic modifications, and TME interactions—allow melanoma cells to escape therapy and support survival under MAPK inhibition [51,52,53,54,55].

To address resistance, rational combination and sequencing strategies are being developed. Dual or triple inhibition of the MAPK pathway, incorporation of ERK inhibitors, and co-targeting of PI3K/AKT or CDK4/6 pathways have shown preclinical efficacy and early clinical promise [56,57,58]. MEK inhibitors combined with CDK4/6 inhibitors can suppress compensatory cell cycle progression, while PI3K/AKT pathway blockade addresses parallel survival signaling. Integration with ICIs is also under investigation. MAPK inhibitors modulate tumor immunogenicity, enhancing antigen presentation and T-cell infiltration, potentially improving the efficacy of ICIs [59,60,61,62]. Biomarker-guided sequencing of these combinations is critical to optimize patient outcomes and limit toxicity [63,64].

In addition to BRAF and MEK mutations, alterations in the KIT oncogene define a distinct molecular subset of melanoma, particularly mucosal and acral types. For these patients, KIT inhibitors such as imatinib (Gleevec) and nilotinib (Tasigna) offer clinically meaningful responses, underscoring the importance of genotype-directed therapy.

Melanoma patients benefit from the off-label use of KIT inhibitors, imatinib and nilotinib, only after stratification based on their KIT mutation status. KIT amplifications do not confer sensitivity to KIT inhibitors. Growing evidence suggest that depending on the melanoma subtype, KIT can act as an oncogene or a tumor suppressor [4].

KIT mutations, present in ~3–8% of melanomas, define a therapeutically relevant subset, especially in acral, mucosal, and chronically sun-damaged subtypes [23,65,66]. Clinical benefit is mutation-dependent: activating KIT point mutations (e.g., L576P, K642E) respond to imatinib or nilotinib, whereas KIT amplifications alone do not. Molecular testing is essential. KIT biology is context-dependent, acting as an oncogene in some subtypes and as a tumor suppressor in others, with implications for therapy selection [67].

KIT inhibitors provide therapeutic options for mucosal, acral, and chronically sun-damaged melanomas with activating KIT mutations in exons 11 and 13. KIT inhibitors show variable responses (16–30%) dependent on genotype, with nilotinib sometimes active post-imatinib [65,66]. Tyrosine kinase inhibitors (TKIs) such as imatinib, sunitinib, and nilotinib achieve objective response rates of 30–50%, though median PFS is often only ~4 months [63,64]. Response depends on specific KIT mutations rather than amplification alone, highlighting the need for comprehensive molecular profiling [68]. Resistance mechanisms include MAPK reactivation, PI3K-AKT pathway compensation, and phenotypic dedifferentiation. Emerging biomarkers, including ctDNA monitoring, may guide therapy sequencing [17,69]. Combination approaches with ICIs or other targeted agents are under active study to improve durability [29,30,70].

A comparison of approved BRAF, MEK, and KIT inhibitors used in the treatment of melanoma is presented in Table 1.

Adverse events vary by drug class. BRAF inhibitors commonly cause arthralgia, rash, fatigue, and photosensitivity [38,39,40]. MEK inhibitors are associated with diarrhea, hypertension, cardiomyopathy, and ocular toxicity [41,42,43,44]. KIT inhibitors frequently induce cytopenias, edema, and hepatotoxicity [63,64]. Early recognition and management of these adverse events are essential for maintaining therapy adherence and quality of life.

BRAF, MEK, and KIT inhibitors illustrate the promise of precision medicine in cutaneous melanoma. They improve ORR, PFS, and OS in molecularly stratified patients, yet resistance and incomplete durability of response remain challenges [31,32,33]. Ongoing research aims to refine combination regimens, develop next-generation inhibitors, and incorporate biomarker-driven strategies to overcome these barriers and optimize long-term outcomes.

In addition to BRAF, MEK, and KIT inhibitors, several other therapeutic modalities are integral to the current management of melanoma. ICIs targeting programmed death-1 (PD-1) (nivolumab, pembrolizumab) and cytotoxic T-lymphocyte–associated protein 4 (CTLA-4) (ipilimumab) constitute the cornerstone of first-line treatment for advanced, unresectable, or metastatic melanoma, providing durable responses and significant survival benefits compared to chemotherapy or monotherapy approaches. Combination ICI therapy with nivolumab and ipilimumab is also approved for selected patients, offering higher response rates but with increased immune-related toxicity [71,72,73].

Oncolytic viral therapy, particularly talimogene laherparepvec (T-VEC), is utilized for unresectable stage IIIB–IVM1a melanoma. T-VEC selectively replicates within tumor cells and expresses granulocyte–macrophage colony-stimulating factor (GM-CSF), enhancing systemic antitumor immunity. It may be administered alone or in combination with ICIs, with ongoing trials demonstrating synergistic activity [74,75].

Prior to the introduction of immunotherapy and targeted agents, cytotoxic chemotherapy (e.g., dacarbazine, temozolomide) was the standard of care for advanced melanoma; however, due to limited efficacy and high toxicity, it is now reserved for refractory or palliative cases [76]. Radiotherapy remains important in the adjuvant or palliative setting, particularly for brain or soft tissue metastases, and may enhance immune activation when combined with checkpoint inhibitors [77].

Surgical resection remains the mainstay of early-stage melanoma management (stages I–II and some stage III), aiming for curative intent. For resectable stage III–IV or high-risk melanoma, adjuvant or neoadjuvant immunotherapy (nivolumab, pembrolizumab) or targeted therapy (dabrafenib plus trametinib in BRAF-mutant cases) are now recommended to reduce recurrence risk and improve long-term survival [78].

Within this therapeutic landscape, BRAF, MEK, and KIT inhibitors are primarily used in patients with BRAF V600– or KIT-mutated melanoma, either as first-line targeted approaches or following immunotherapy failure, depending on molecular profile, tumor burden, and clinical course. These treatment strategies collectively illustrate the evolution of melanoma therapy, summarized in Table 2, which provides an overview of melanoma treatment strategies, classified according to therapeutic modality and summarizes the most severe and frequent adverse events, including their severity.

## 3. Vemurafenib—BRAF Inhibitor

Vemurafenib is a small-molecule, ATP-competitive kinase inhibitor developed through fragment-based drug discovery and designed to selectively target BRAFV600E/K mutations, which occur in approximately 40–60% of cutaneous melanomas [79,80]. These gain-of-function mutations cause constitutive activation of the mitogen-activated protein kinase (MAPK)/extracellular signal-regulated kinase (ERK) signaling pathway, independent of upstream RAS activity, thereby driving uncontrolled proliferation, enhanced survival, and resistance to apoptosis.

**Figure 1:**
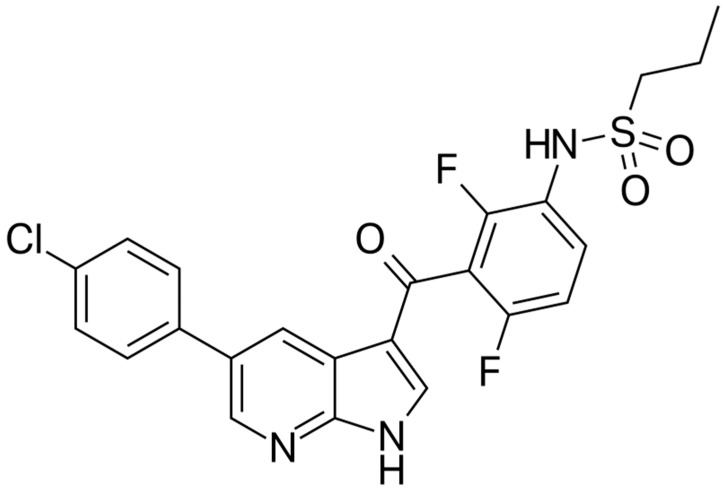
Structural formula of vemurafenib.

Figure 1 illustrates the chemical structure of vemurafenib.

The therapeutic effect of vemurafenib arises from its selective binding to mutant BRAF monomers, inhibiting downstream phosphorylation of MEK and ERK. This suppression of oncogenic MAPK signaling induces G1-phase cell cycle arrest, promotes apoptosis, and leads to marked tumor regression in melanomas harboring BRAFV600E/K alterations [81,82,83,84].

**Figure 2:**
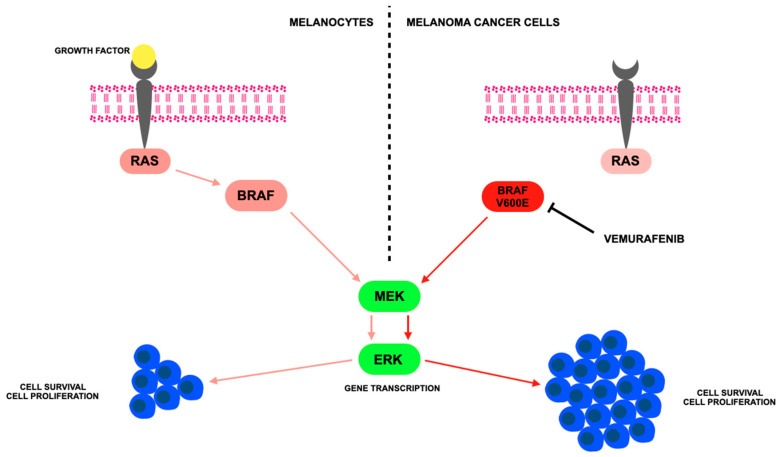
The mechanism of vemurafenib according to [85] involves targeting the MAPK/ERK signaling pathway, specifically inhibiting the BRAF V600E mutation—where valine (V) at position 600 is replaced by glutamic acid (E)—which causes continuous activation of the BRAF protein and promotes cancer growth; where: ERK—Extracellular Signal-Regulated Kinase, a protein kinase activated by MEK that controls cell division, survival, and differentiation; MEK—Mitogen-Activated Protein Kinase Kinase, an enzyme that activates ERK by phosphorylation and acts downstream of RAS and BRAF in the signaling pathway; RAS—a family of proteins (e.g., KRAS, NRAS, HRAS) that regulate cell growth and survival by transmitting signals from outside the cell to the nucleus; mutations can lead to cancer.

Figure 2 illustrates the mechanism of action of vemurafenib.

Preclinical studies, employing both in vitro cellular assays and in vivo xenograft models, demonstrated that vemurafenib exhibits high specificity and potent antitumor activity against melanoma cells carrying the BRAFV600E mutation. These findings provided strong mechanistic and pharmacological rationale for its rapid progression into clinical development. Early-phase clinical trials, initiated in 2008, confirmed the preclinical evidence, showing substantial tumor regression and ORRs ranging from 50% to 80% in patients with advanced BRAF-mutant melanoma. Further validation came from the pivotal BRIM-3 randomized, multicenter phase III trial, which compared vemurafenib with dacarbazine in previously untreated patients with metastatic BRAF-mutant melanoma. The trial was terminated early following the demonstration of a significant survival benefit in the vemurafenib group. Specifically, vemurafenib reduced the risk of death by 63% compared to dacarbazine (hazard ratio [HR] = 0.37; *p* < 0.0001) and significantly improved both PFS and OS. These results led to its accelerated approval by the U.S. Food and Drug Administration (FDA) in 2011 and the European Medicines Agency (EMA) in 2012 [86,87].

Beyond melanoma, the clinical application of vemurafenib expanded in 2017 with its approval for Erdheim–Chester disease (ECD), a rare non-Langerhans cell histiocytosis characterized by BRAFV600E mutations. In this setting, vemurafenib has produced durable clinical responses, radiographic tumor regression, and substantial symptom relief, highlighting the oncogenic relevance of BRAFV600E across diverse malignancies [88,89].

Table 3 summarizes the distribution and clinical significance of RAF mutations across different cancer types.

In routine clinical practice, vemurafenib has significantly reshaped treatment expectations for patients with advanced BRAF-mutant melanoma. Compared with dacarbazine, it doubled median PFS from 1.6 to approximately 5.3 months and achieved ORRs of 50–60%, often inducing rapid and profound tumor regression [91,92,93]. However, the durability of these responses is limited, with most patients experiencing disease progression within six to eight months.

Mechanisms of resistance to vemurafenib are diverse and multifactorial. Reactivation of the MAPK pathway is the most frequent, often mediated by secondary NRAS mutations that promote RAF dimerization, bypassing the monomer-selective inhibition of vemurafenib. Alternative BRAF splicing, particularly the p61BRAF isoform, facilitates constitutive dimerization and drug evasion, while BRAF amplification can elevate protein levels beyond the inhibitory threshold. Upregulation of RTKs such as PDGFRβ, IGF-1R, and EGFR, and activation of compensatory survival signaling via the PI3K–AKT pathway—particularly in the context of PTEN loss—further contribute to resistance. The tumor microenvironment also plays a pivotal role; stromal fibroblasts and tumor-associated macrophages can secrete hepatocyte growth factor, activating c-MET signaling and reinforcing resistance phenotypes [94,95,96,97].

Vemurafenib is generally well tolerated, with common adverse events including arthralgia, rash, photosensitivity, and fatigue, which are typically reversible with supportive care or dose adjustment. More serious but less frequent toxicities include hepatotoxicity, QT interval prolongation, and bradyarrhythmias, which warrant biochemical and electrocardiographic monitoring. Additional adverse events include gastrointestinal disturbances, musculoskeletal pain, secondary cutaneous neoplasms with an incidence of approximately 12%, and rare complications such as uveitis and cytochrome P450–mediated drug interactions [98,99,100]. Toxic epidermal necrolysis has been reported in combination with cobimetinib [101]. A clinically significant adverse event is the development of cutaneous squamous cell carcinomas and keratoacanthomas, observed in 20–30% of treated patients. These low-grade lesions, usually managed by surgical excision, arise from paradoxical MAPK activation in RAS-mutant, BRAF–wild-type keratinocytes, representing an on-target, off-tumor effect of vemurafenib [102,103].

To overcome resistance and enhance response durability, dual inhibition of the MAPK pathway with vemurafenib and MEK inhibitors such as cobimetinib or trametinib has demonstrated superior outcomes. These regimens prolong median PFS to approximately 12 months, compared with around seven months for monotherapy, and reduce paradoxical cutaneous toxicities by suppressing MEK-driven signaling in non-malignant cells [22,104]. Building on this, triplet regimens incorporating BRAF and MEK inhibitors with ICIs are under investigation. The IMspire150 trial showed that the addition of atezolizumab (anti–PD-L1) to vemurafenib and cobimetinib significantly improved PFS and overall survival in advanced BRAF-mutant melanoma, supporting the hypothesis that targeted therapies can enhance neoantigen release and improve immune checkpoint blockade efficacy [105,106,107,108,109].

Emerging strategies aim to delay or overcome resistance through inhibitors of the PI3K, AKT, and mTOR pathways to block compensatory survival signals, histone deacetylase inhibitors to reverse transcriptional reprogramming, and HSP90 inhibitors to degrade mutant BRAF and other oncogenic proteins. Immunomodulatory agents are being explored to remodel the tumor microenvironment, enhance antigen presentation, and facilitate T-cell infiltration. Additional targets include integrins and TGF-β signaling components such as ITGA5, ITGB3, PAI1, and p21, which may provide new avenues for overcoming resistance and improving therapeutic outcomes [110,111,112,113,114].

Future directions focus on optimizing vemurafenib therapy through intermittent dosing to reduce selective pressure and toxicity, advanced drug delivery systems such as nanoparticles and liposomes to improve pharmacokinetics and minimize off-target effects, and real-time biomarker monitoring using circulating tumor DNA, phosphoproteomics, and single-cell RNA sequencing to enable adaptive treatment strategies [115,116,117]. Vemurafenib has redefined the therapeutic landscape for BRAF-mutant melanoma and exemplifies the clinical potential of mutation-targeted drug development. Its success has catalyzed the adoption of molecularly guided therapeutic strategies across oncology. Continued innovation through rational drug combinations, integration of dynamic biomarkers, and development of next-generation therapeutic platforms holds promise for achieving durable disease control, reducing toxicity, and moving closer to long-term remission in this historically aggressive malignancy [118,119,120,121,122,123,124,125,126,127,128,129,130,131].

## 4. Trametinib—MEK Inhibitor

Trametinib is a selective, reversible, allosteric inhibitor of mitogen-activated protein kinase kinases 1 and 2 (MEK1/2), key downstream components of the RAS–RAF–MEK–ERK signaling cascade, also known as the mitogen-activated protein kinase (MAPK) pathway. Dysregulation of this pathway is a hallmark of multiple malignancies, with particular pathogenic importance in cutaneous melanoma. The MAPK cascade regulates critical cellular processes such as proliferation, differentiation, survival, and migration. In melanoma, activating BRAF mutations—most notably BRAFV600E and BRAFV600K—drive constitutive MEK activation and sustained ERK phosphorylation, leading to aberrant oncogenic signaling independent of upstream receptor stimulation or external mitogenic cues [132,133].

**Figure 3:**
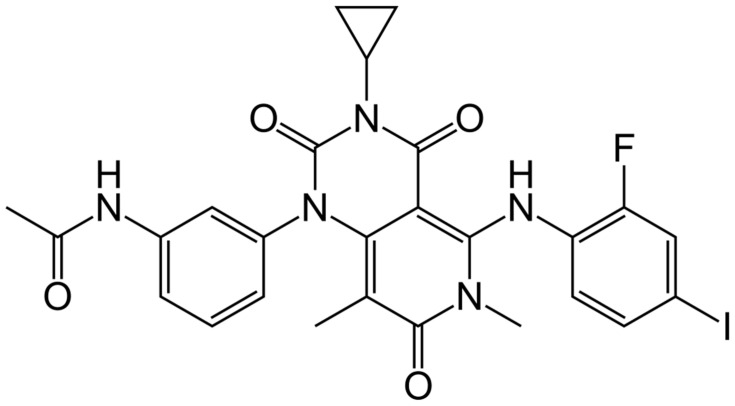
Structural formula of trametinib.

Figure 3 illustrates the chemical structure of trametinib.

Mechanistically, trametinib binds to MEK1/2 in a non-competitive, allosteric manner adjacent to the ATP-binding site, stabilizing the kinase in its inactive conformation and preventing phosphorylation by upstream RAF kinases. This inhibition results in potent suppression of ERK phosphorylation (pERK), downregulation of proliferation- and survival-associated gene expression, G1-phase cell cycle arrest, and induction of apoptosis in sensitive tumor cells [134,135]. The mechanism is particularly relevant in BRAFV600-mutant cancers, where persistent MAPK pathway activation sustains oncogenic growth.

**Figure 4:**
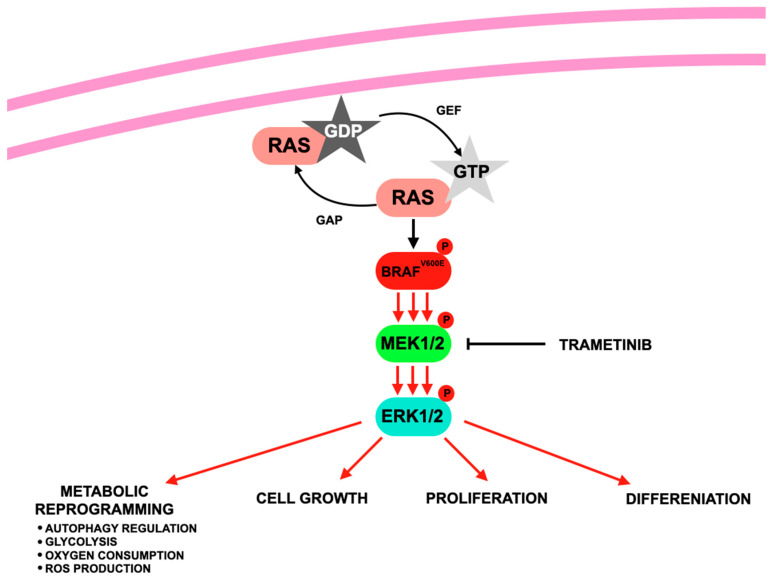
Mechanism of trametinib according to [136] involves inhibition of the BRAF–MEK1/2–ERK1/2 signaling pathway, which is activated when RAS, a small GTPase, switches from an inactive GDP-bound form to an active GTP-bound form, initiating a phosphorylation cascade wherein mutated BRAF (e.g., V600E) activates MEK, which subsequently phosphorylates and activates ERK, ultimately promoting metabolic reprogramming, cell growth, proliferation, and differentiation; where: BRAF—a protein kinase; MEK—Mitogen-Activated Protein Kinase Kinase; ERK—Extracellular Signal-Regulated Kinase; RAS—a small GTPase; GAP—GTPase-activating protein; GEF—Guanine nucleotide exchange factor; GDP—guanosine diphosphate; GTP—guanosine triphosphate.

Figure 4 illustrates the mechanism of action of trametinib.

Trametinib became the first MEK inhibitor to achieve regulatory approval, marking a significant milestone in targeted oncology. In May 2013, the U.S. Food and Drug Administration (FDA) approved trametinib monotherapy for patients with unresectable or metastatic melanoma harboring BRAFV600E or BRAFV600K mutations, based on phase III trials demonstrating improved PFS and OS compared with conventional chemotherapy such as dacarbazine [94,137,138]. Unlike BRAF inhibitors, trametinib does not cause paradoxical MAPK activation in RAS-mutant keratinocytes, reducing the risk of secondary cutaneous neoplasms.

Despite its initial clinical efficacy, resistance to trametinib monotherapy frequently develops within four to six months. The mechanisms underlying resistance include activation of compensatory survival pathways such as PI3K/AKT/mTOR, secondary MEK1 mutations (e.g., MEK1P124L/Q) that reduce drug binding affinity, and upregulation of RTKs including PDGFRβ and IGF-1R, which reactivate MAPK signaling through bypass mechanisms [94,139].

To overcome resistance and improve treatment outcomes, trametinib is often combined with the BRAF inhibitor dabrafenib. This dual blockade of the MAPK pathway provides synergistic inhibition, limits feedback reactivation, and delays the emergence of resistance. The pivotal COMBI-d and COMBI-v phase III trials demonstrated significantly improved outcomes with the combination compared with dabrafenib monotherapy, including median PFS of 11.0 versus 8.8 months, overall survival of 25.1 versus 18.7 months, and objective response rates of 67% versus 51% [140,141,142,143,144,145,146,147,148,149,150,151]. Subsequent studies confirmed these findings across diverse populations, including Chinese patients with resected stage III disease [152] and pediatric and adolescent cohorts [153]. These data established the dabrafenib–trametinib combination as the standard of care for advanced BRAF-mutant melanoma and stimulated further exploration of dual MAPK inhibition in other malignancies [154,155,156,157].

The safety profile of trametinib reflects its mechanism of MEK inhibition in non-malignant tissues. Common adverse events include acneiform dermatitis, diarrhea, peripheral edema, and hypertension, consistent with effects on epithelial and vascular signaling [158,159,160]. More serious but less frequent toxicities include cardiomyopathy, manifesting as decreased left ventricular ejection fraction, and ocular events such as central serous retinopathy and retinal vein occlusion, necessitating cardiovascular and ophthalmologic monitoring [161,162]. Importantly, trametinib does not induce cutaneous squamous cell carcinomas—a toxicity observed with BRAF inhibitors—owing to its upstream inhibition that prevents paradoxical ERK activation in RAS-mutant keratinocytes [41].

In efforts to enhance therapeutic durability and immune engagement, rational triplet regimens have been developed. Clinical trials combining dabrafenib, trametinib, and ICIs—including spartalizumab (anti–PD-1) and atezolizumab (anti–PD-L1)—have shown encouraging results. Proposed mechanisms of synergy involve increased T-cell infiltration, suppression of regulatory T cells and myeloid-derived suppressor cells, and improved antigen presentation within the tumor microenvironment [163,164,165]. The IMspire150 phase III trial, using a similar triplet of vemurafenib, cobimetinib, and atezolizumab, further supports the therapeutic potential of MEK inhibitor–based triplet combinations [166].

Beyond melanoma, trametinib has demonstrated clinical efficacy across multiple BRAFV600-mutant malignancies, including non-small cell lung cancer, anaplastic thyroid carcinoma, and histiocytic disorders such as Erdheim–Chester disease and Langerhans cell histiocytosis [167,168]. Ongoing research is exploring its combination with inhibitors of compensatory signaling pathways, including PI3K, HDAC, and HSP90 inhibitors, to overcome resistance and broaden its therapeutic scope. Basket trials are also assessing trametinib in genetically defined malignancies harboring MAPK pathway alterations [169,170,171,172,173,174,175,176,177].

Preclinical and translational research has highlighted trametinib’s immunomodulatory properties. MEK inhibition can remodel the tumor microenvironment by suppressing pro-tumorigenic cytokine production, enhancing cytotoxic T-cell function, and reducing immunosuppressive cell populations. These immune-modifying effects may potentiate responses to immune checkpoint blockade, positioning trametinib as a potential immune-sensitizing agent in refractory cancers [178,179,180]. Clinical trials are underway to evaluate its integration into combination regimens for checkpoint inhibitor–resistant malignancies [15,181,182,183].

Overall, trametinib represents a cornerstone of targeted therapy for MAPK-driven cancers, particularly in combination with dabrafenib for BRAF-mutant melanoma. Its incorporation into multidrug regimens exemplifies a mechanism-based therapeutic strategy designed to enhance treatment depth, delay resistance, and improve immune responsiveness. As precision oncology continues to evolve toward more personalized and durable treatment paradigms, trametinib remains a key agent in the expanding landscape of cancer therapeutics.

## 5. Imatinib—C-KIT Inhibitor

Imatinib mesylate is a first-in-class, small-molecule TKI that selectively targets the ATP-binding sites of several oncogenic kinases, most notably BCR-ABL, c-KIT (CD117), and platelet-derived growth factor receptors (PDGFR) α and β. These kinases are critical regulators of intracellular signaling pathways controlling cellular proliferation, differentiation, survival, and apoptosis [184,185,186].

**Figure 5:**
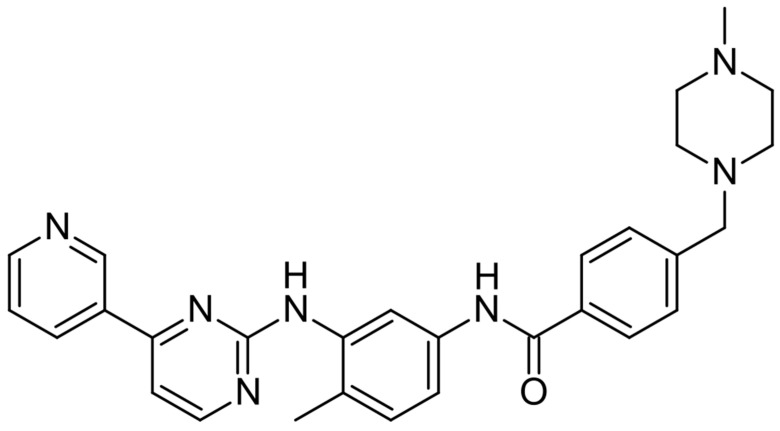
Structural formula of imatinib.

Figure 5 illustrates the molecular structure of imatinib.

Mechanistically, imatinib binds competitively to the ATP-binding pocket of these kinases in their inactive conformation, preventing autophosphorylation and subsequent phosphorylation of downstream substrates. This blockade effectively disrupts aberrant signal transduction pathways responsible for malignant transformation and uncontrolled cellular growth. The high target selectivity conferred by this mechanism minimizes off-target interactions commonly associated with conventional cytotoxic chemotherapies, thereby contributing to its favorable therapeutic index [187,188,189].

The development of imatinib mesylate marked a paradigm shift in oncology, originating from its ability to selectively inhibit the constitutively active BCR-ABL fusion protein—an aberrant tyrosine kinase generated by the t(9;22)(q34;q11) translocation known as the Philadelphia chromosome. This cytogenetic abnormality defines chronic myeloid leukemia (CML) and drives unchecked proliferation and impaired apoptosis in hematopoietic progenitor cells [190,191,192]. Preclinical studies demonstrated that imatinib induces apoptosis and G1-phase cell cycle arrest in BCR-ABL–expressing cells, providing strong rationale for clinical translation [184]. Following these findings, early 1990s research led by Druker and colleagues developed the first potent and specific BCR-ABL inhibitor, rapidly advancing it into clinical trials. Early-phase studies showed unprecedented hematologic and cytogenetic responses in interferon-refractory CML, leading to accelerated approval in 2001 and ushering in the modern era of precision oncology [193,194,195].

Approved in 2001, imatinib revolutionized the treatment of CML, transforming a once-fatal disease into a chronic, manageable condition characterized by dramatic improvements in progression-free and overall survival. Beyond CML, imatinib demonstrated substantial clinical efficacy in gastrointestinal stromal tumors (GIST), which frequently harbor activating mutations in c-KIT or PDGFRA that lead to ligand-independent receptor activation and oncogenic signaling. Imatinib effectively inhibits these mutant kinases, resulting in tumor regression and prolonged survival in patients with advanced or metastatic GIST [196,197,198,199]. These successes established imatinib as a prototype molecularly targeted agent and validated the strategy of exploiting discrete oncogenic dependencies across tumor types [200,201,202,203].

Imatinib is FDA-approved for a broad range of malignancies driven by dysregulated kinase activity, including Philadelphia chromosome–positive CML and acute lymphoblastic leukemia (ALL), KIT-mutant GIST, myelodysplastic/myeloproliferative disorders with PDGFR rearrangements, dermatofibrosarcoma protuberans, hypereosinophilic syndrome (HES), chronic eosinophilic leukemia, and aggressive systemic mastocytosis lacking the KIT D816V mutation. Off-label uses include chordomas, desmoid tumors, advanced KIT-mutant melanoma, and post–allogeneic transplant relapse in CML [204,205,206,207].

In melanoma, imatinib has shown modest but clinically meaningful efficacy in specific biologically distinct subtypes characterized by KIT mutations or amplifications, including acral, mucosal, and chronically sun-damaged melanomas [208,209,210]. While the overall response rates are lower than in CML or GIST, partial responses and durable disease stabilization have been observed in selected patients, underscoring the importance of molecular stratification [26,65]. Clinical studies specifically evaluating KIT-mutant acral, mucosal, uveal, and sun-damaged melanomas have reported heterogeneous yet encouraging results [211,212,213,214,215,216].

Despite its transformative success, resistance to imatinib frequently emerges, particularly in advanced stages of disease. Mechanisms of resistance include point mutations within the BCR-ABL kinase domain—most notably the T315I mutation—gene amplification, and activation of alternative survival pathways [217,218,219,220]. To overcome these limitations, second- and third-generation TKIs such as dasatinib, nilotinib, bosutinib, and ponatinib were developed, providing enhanced potency and broader activity against resistant BCR-ABL variants [221,222,223,224].

Imatinib exhibits a favorable safety profile that supports long-term use. The most common adverse events are mild and include fluid retention, nausea, myalgia, fatigue, and low-grade myelosuppression [225,226]. Severe but rare toxicities include hepatotoxicity, cardiotoxicity, and prolonged cytopenias, necessitating regular monitoring [226,227]. The overall tolerability of imatinib contributes to its sustained use in chronic disease management such as CML [228].

Emerging research continues to expand the therapeutic applications of imatinib through advanced molecular profiling, identifying novel tumors with imatinib-sensitive kinase alterations. In melanoma, early intervention in KIT-mutant acral or mucosal subtypes appears to yield superior outcomes [26,229]. Combination strategies incorporating imatinib with immune checkpoint inhibitors or other targeted agents are actively being investigated to suppress compensatory pathways, overcome resistance, and enhance therapeutic durability [230,231,232,233,234,235,236,237,238,239]. Parallel developments include next-generation TKIs, allosteric inhibitors, and targeted protein degraders designed to inhibit or eliminate oncogenic kinases more effectively [218,240,241].

Imatinib’s discovery and clinical success remain a landmark in oncology, exemplifying the transformative impact of rational drug design and molecularly guided therapy on patient outcomes [242,243,244]. In melanoma, it continues to serve as a valuable therapeutic option for patients with KIT mutations or amplifications, with ongoing research focused on refining patient selection, optimizing combination regimens, and integrating imatinib into evolving immunotherapy and precision oncology frameworks [55,65].

**Figure 6:**
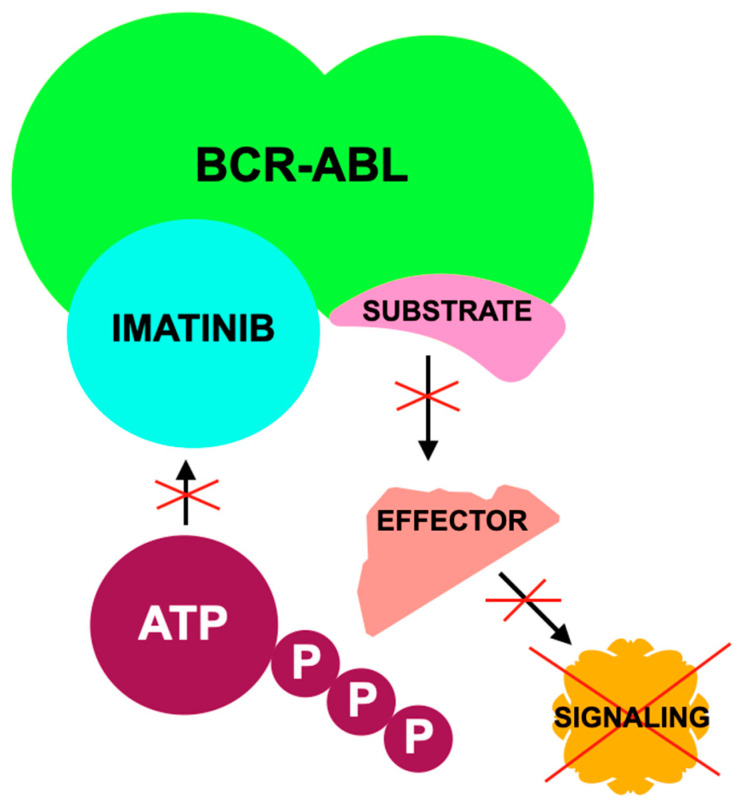
The mechanism of imatinib according to [245] involves inhibition of the BCR-ABL fusion protein, a constitutively active tyrosine kinase, by occupying its ATP-binding site; this prevents substrate phosphorylation, disrupts downstream effector interactions, interrupts the signal transduction pathway, and ultimately blocks oncogenic signaling, halts uncontrolled cell proliferation, and induces apoptosis, where: ATP—adenosine triphosphate; P—phosphate.

Figure 6 illustrates molecular mechanism underlying imatinib activity.

## 6. Comparative Analysis of Vemurafenib, Trametinib, and Imatinib in Cutaneous Melanoma

Targeted therapies have markedly improved outcomes for patients with BRAF- or KIT-mutant cutaneous melanoma, yet differences in mechanism, efficacy, and toxicity necessitate a comparative evaluation of vemurafenib, trametinib, and imatinib. Vemurafenib is a small-molecule, ATP-competitive inhibitor that selectively targets BRAFV600E/K mutations, present in 40–60% of cutaneous melanomas, thereby blocking constitutive activation of the MAPK/ERK pathway and inducing G1-phase arrest, apoptosis, and tumor regression [65,66,67,68,69,70]. Preclinical studies demonstrated potent, mutation-specific antitumor activity, providing the rationale for rapid clinical development. Early-phase trials initiated in 2008 showed ORRs of 50–80% in advanced BRAF-mutant melanoma. The pivotal BRIM-3 phase III trial confirmed a median PFS of 5.3 months versus 1.6 months with dacarbazine and a median OS of 13.6 months versus 9.7 months, leading to FDA approval in 2011 and EMA approval in 2012 [86,87]. Combination therapy with MEK inhibitors such as trametinib or cobimetinib further improved outcomes, extending median PFS to approximately 12 months and increasing ORRs to ~70% while reducing paradoxical MAPK-driven toxicities [22,104]. Triplet regimens including ICIs, such as atezolizumab, are under investigation and show promise for enhancing neoantigen-mediated immune responses [106,107,108,109]. Common adverse events for vemurafenib include arthralgia, rash, photosensitivity, fatigue, gastrointestinal disturbances, hepatotoxicity, and QT prolongation, with more serious but less frequent events including cutaneous squamous cell carcinoma or keratoacanthomas (~20–30%) [97,98,99,100,101,102]. Resistance mechanisms are multifactorial and include MAPK reactivation via secondary NRAS or MEK mutations, BRAF amplification or alternative splicing, upregulation of RTKs (PDGFRβ, IGF-1R, EGFR), compensatory PI3K–AKT signaling, and tumor microenvironment interactions [110,111,112,113,114].

Trametinib is a selective MEK1/2 inhibitor acting downstream of BRAF to prevent ERK phosphorylation and inhibit cellular proliferation. Monotherapy trials reported ORRs of 20–25%, median PFS of 4–5 months, and median OS of approximately 11 months [54,55,56]. However, when combined with BRAF inhibitors, trametinib significantly improves ORRs to 60–70%, median PFS to 11–12 months, and OS up to 25 months, demonstrating superior durability of response compared with monotherapy [22,104]. Adverse events include rash, diarrhea, fatigue, peripheral edema, hypertension, cardiomyopathy, and retinopathy, with rare cases of interstitial lung disease [41,42,43]. Resistance to trametinib commonly arises through MEK or ERK mutations, receptor tyrosine kinase upregulation, or bypass signaling, and current investigational strategies include ERK inhibitors, PI3K/AKT pathway antagonists, CDK4/6 inhibitors, and combination with ICIs to enhance anti-tumor immunity [110,111,112,113,114].

Imatinib, a tyrosine kinase inhibitor targeting KIT mutations, is primarily used in acral, mucosal, or chronically sun-damaged melanomas harboring activating KIT alterations, particularly in exons 11 and 13. Preclinical studies demonstrated potent inhibition of KIT-mutant melanoma cells. Phase II clinical trials showed ORRs of 30–50%, median PFS of approximately 3.5–4 months, and median OS around 12 months [25,26,27,28]. Responses are largely limited to patients with specific KIT mutations, with resistance frequently developing within months. Adverse effects are generally mild to moderate, including edema, nausea, diarrhea, fatigue, and rash, while severe toxicities such as myelosuppression, hepatotoxicity, and cardiotoxicity have been reported [63,64]. Combination strategies with ICIs or chemotherapy are under investigation to enhance efficacy and overcome resistance [29,30].

Comparing these therapies, BRAF-targeted combination therapy (vemurafenib plus trametinib) demonstrates the highest clinical efficacy, with OS up to 25 months, PFS approximately 12 months, and ORRs of 60–70%. Vemurafenib monotherapy offers moderate efficacy with OS of ~13.6 months, PFS of ~5.3 months, and ORRs of 50–60%, while imatinib shows intermediate ORRs (30–50%) and lower durability, primarily in KIT-mutant melanoma. Resistance remains a challenge across all agents, driven by genetic and non-genetic mechanisms, necessitating combination therapy, careful monitoring, and biomarker-guided patient selection. Toxicity profiles differ: vemurafenib is associated with cutaneous events and photosensitivity, trametinib with rash and cardiotoxicity, and imatinib with fluid retention and cytopenias. Integration of ICIs with BRAF/MEK inhibitors is emerging as a strategy to enhance durability and achieve long-term disease control [105,106,107,108,109].

In conclusion, vemurafenib/trametinib combination therapy provides the most effective treatment for BRAF-mutant melanoma, while vemurafenib monotherapy and imatinib remain important options for selected patient subsets. Future research focusing on resistance mechanisms, rational combination strategies, and biomarker-driven personalization is expected to further improve outcomes and advance the precision management of cutaneous melanoma.

Table 4 presents a comparative analysis of the mechanisms of action of vemurafenib, trametinib, and imatinib, along with their combination strategies and associated clinical biomarkers and Table 5 illustrates a comparison of adverse effects of vemurafenib, trametinib, and imatinib.

## 7. Future Directions and Clinical Relevance

The therapeutic landscape of cutaneous melanoma has been revolutionized by targeted agents such as vemurafenib, trametinib, and imatinib, which have substantially improved outcomes for patients with advanced disease. Despite these advances, significant clinical challenges persist, including the emergence of drug resistance, limited durability of responses, intra- and inter-tumoral heterogeneity, and the need for individualized treatment strategies. Consequently, current research focuses on refining therapeutic approaches, overcoming resistance mechanisms, and integrating targeted therapies with complementary treatment modalities to achieve durable disease control and improved long-term survival.

One of the most pressing challenges in targeted melanoma therapy is the development of resistance to BRAF, MEK, and KIT inhibitors. Resistance arises through various mechanisms, including secondary mutations, activation of compensatory signaling pathways such as PI3K/AKT/mTOR, or phenotypic switching toward a mesenchymal-like state that promotes therapeutic escape [247]. For example, reactivation of the MAPK pathway downstream of BRAF via MEK or ERK mutations has been documented in patients progressing on BRAF/MEK inhibitors, underscoring the adaptive plasticity of melanoma cells [235]. Novel approaches to circumvent resistance are under investigation, including dual or triple blockade of the MAPK pathway, co-targeting of parallel signaling networks, and inhibition of RTKs that drive adaptive survival responses [248,249]. These strategies aim to suppress compensatory mechanisms, extend response duration, and prevent or delay resistance development.

Combination therapy is another critical area of ongoing investigation. The rationale for combining targeted therapies with immunotherapies stems from their complementary mechanisms of action: targeted agents induce rapid tumor regression and modulate the tumor microenvironment, while immune checkpoint inhibitors (ICIs) provide durable immune surveillance and control. Clinical trials combining BRAF/MEK inhibitors with PD-1 or PD-L1 blockade have shown improved progression-free survival and durable responses compared with monotherapies [250,251]. Innovative strategies integrating targeted therapy with adoptive T-cell transfer, oncolytic virus therapy, or personalized cancer vaccines are also being explored, aiming to enhance antitumor immunity and achieve more robust clinical outcomes [252,253]. These multimodal approaches represent a paradigm shift in melanoma management, moving from sequential monotherapies to integrated treatment models that exploit synergistic mechanisms.

Precision oncology is increasingly guiding clinical decision-making, enabling individualized therapy selection based on molecular profiling. Advances in next-generation sequencing (NGS), liquid biopsy technologies, and circulating tumor DNA (ctDNA) analysis now allow for real-time monitoring of tumor evolution and treatment response [254]. These tools facilitate early detection of resistance-associated mutations, inform dynamic treatment adjustments, and enable adaptive therapy before clinical progression [255]. Moreover, multi-omics integration—including transcriptomic and proteomic profiling—is expected to uncover predictive biomarkers of therapeutic response and resistance, thereby refining patient stratification and optimizing treatment selection [256].

The development of next-generation inhibitors remains a key focus of translational research. Novel pan-RAF inhibitors, ERK inhibitors, and allosteric MEK inhibitors are being investigated to overcome resistance to first-generation agents and broaden activity against resistant clones [257]. Similarly, new KIT inhibitors with improved potency and selectivity are under evaluation for KIT-mutant melanoma, a rare but challenging subtype with limited treatment options [258,259]. These compounds may become important components of rational combination regimens or serve as salvage therapies following disease progression on standard treatments.

As the therapeutic landscape evolves, real-world application of these strategies will require careful consideration of toxicity management, treatment sequencing, and cost-effectiveness. The integration of molecular diagnostics into routine clinical workflows demands robust bioinformatics infrastructure and multidisciplinary collaboration among oncologists, pathologists, geneticists, and data scientists. Ensuring equitable access to advanced diagnostics and therapies remains a critical global priority, particularly in resource-limited healthcare systems [260].

The next decade of melanoma therapy is poised to witness the convergence of targeted therapy, immunotherapy, and precision medicine, ushering in a new era of personalized oncologic care. The future relevance of targeted agents such as vemurafenib, trametinib, and imatinib will likely be defined not as standalone treatments but as integral components of multifaceted, patient-specific strategies. Liquid biopsy-guided adaptive therapy, multi-omics-driven biomarker discovery, and the integration of artificial intelligence for predictive modeling are expected to further refine treatment selection and monitoring [254,256]. Emerging approaches—including personalized neoantigen vaccines, adoptive T-cell therapies, and epigenetic modulators—offer additional avenues to enhance the efficacy of targeted interventions [252,255]. As these innovations mature, the overarching goal will shift from merely extending survival to achieving durable remission and, ultimately, functional cures in select patient populations.

Collectively, these developments underscore a rapidly evolving therapeutic landscape in cutaneous melanoma—one defined by innovation, integration, and personalization. The continued refinement of targeted therapies, in conjunction with advances in molecular diagnostics and immunotherapy, promises to extend the boundaries of clinical benefit. As the field moves toward adaptive, data-driven treatment paradigms, the emphasis is shifting from short-term tumor control to sustained remission and improved quality of life. The following conclusion synthesizes the key insights from this review and highlights their clinical relevance, emphasizing how ongoing translational progress is reshaping both current practice and future research directions in melanoma management.

Table 6 summarizes novel melanoma therapies, classified based on their mechanisms of action and therapeutic strategies.

## 8. Conclusions

The advent of targeted therapies such as vemurafenib, trametinib, and imatinib has transformed the management of cutaneous melanoma, enabling precision treatment guided by molecular profiling. These agents exemplify the success of genotype-driven therapy, demonstrating substantial improvements in survival and quality of life for patients with BRAF or c-KIT mutations. However, their long-term efficacy remains constrained by acquired resistance and cumulative toxicity, emphasizing the need for ongoing molecular and clinical investigation.

Future research should prioritize elucidating resistance mechanisms, refining predictive biomarkers, and developing next-generation inhibitors with improved selectivity and safety. Integrating targeted therapies with immune checkpoint inhibitors and other novel modalities holds promise for enhancing the durability of the response and overcoming therapeutic escape.

Sustained collaboration across translational research, drug development, and clinical practice is essential to extend treatment durability and achieve more personalized, durable outcomes for patients with cutaneous melanoma.

## Figures and Tables

**Table 1:** Approved BRAF, MEK, and KIT inhibitors for melanoma according to [15,16,17,23,45]. All abbreviations employed are defined in the text in the Abbreviations section.

Class/ Agent	Key Targets	Pivotal Trials	ORR (%)	Median PFS (mo)	Median OS (mo)	Common AEs	Distinct Features/ Resistance Notes
Vemurafenib (Zelboraf)	BRAF V600E/K	BRIM-3	48	6.9	13.6	Photosensitivity, arthralgia	First-in-class; higher cutaneous toxicity; paradoxical MAPK activation
Dabrafenib (Tafinlar)	BRAF V600E/K	BREAK-3	51	9.3	18.2	Pyrexia, fatigue	Lower rash/photosensitivity; improved tolerability
Encorafenib (Braftovi)	BRAF V600E/K	COLUMBUS	60	14.9	33.6	Arthralgia, fatigue	Longest half-life; least paradoxical activation
Trametinib (Mekinist)	MEK1/2	METRIC	22	4.8	15.6	Rash, diarrhea	Alone or combined with dabrafenib
Cobimetinib (Cotellic)	MEK1	coBRIM	— (combo)	12.3	22.3	Diarrhea, photosensitivity	Used with vemurafenib
Binimetinib (Mektovi)	MEK1/2	COLUMBUS	— (combo)	14.9	33.6	Fatigue, nausea	Used with encorafenib
Imatinib (Gleevec)	KIT (L576P, K642E)	Phase II	16–29	3–7	12–18	Edema, nausea	Benefit limited to activating mutations; not amplifications
Nilotinib (Tasigna)	KIT (L576P, K642E)	Phase II	20–26	3–6	—	Fatigue, cytopenia	May retain efficacy post-imatinib; off-label use

**Table 2:** Melanoma Treatment Strategies—overview by modality according to [14,72,74], where: BRAF—v-Raf murine sarcoma viral oncogene homolog B; CTLA-4—Cytotoxic T-lymphocyte–associated protein 4; HSV—Herpes simplex virus; IFN-γ—Interferon gamma; LDH—Lactate dehydrogenase; MAPK—Mitogen-activated protein kinase; MEK—MAPK/ERK kinase; MHC—Major histocompatibility complex; NGS—Next-generation sequencing; NRAS—Neuroblastoma RAS viral oncogene homolog; PD-1—Programmed cell death protein 1; PD-L1—Programmed death-ligand 1; PCR—Polymerase chain reaction; QT—QT interval, RTK—Receptor tyrosine kinase; SLNB—Sentinel lymph node biopsy; TME—Tumor microenvironment; TMB—Tumor mutational burden.

Treatment Type	Drug/ Class	Molecular Target/ Mechanism	Indications	Biomarkers	Combination Strategies	Common Resistance Mechanisms	Key Side Effects (Including Severe/Fatal)
Targeted Therapy	Vemurafenib, Dabrafenib	BRAF V600E/K mutation, inhibits MAPK pathway at BRAF	BRAF V600+ advanced or metastatic melanoma	BRAF V600 mutation (PCR/NGS)	With MEK inhibitors (e.g., Trametinib, Cobimetinib)	BRAF amplification, NRAS mutation, MAPK reactivation	Rash, arthralgia, QT prolongation (potentially fatal arrhythmia), photosensitivity, cutaneous squamous cell carcinoma (severe)
	Trametinib, Cobimetinib	MEK1/2 inhibition, downstream of BRAF	BRAF V600+ (always combined with BRAF inhibitors)	BRAF mutation	With BRAF inhibitors to avoid resistance	MEK/ERK mutations, RTK upregulation	Diarrhea, fatigue, cardiac toxicity (heart failure, rare fatal), retinopathy, interstitial lung disease (rare severe)
	KIT inhibitors (Imatinib, Nilotinib)	KIT mutations or amplifications (rare subtype of melanoma)	Acral, mucosal, or chronically sun-damaged melanoma	KIT exon 11/13 mutation	Rarely used; sometimes with immunotherapy or chemotherapy	Secondary KIT mutations	Edema, cytopenias, nausea, hepatotoxicity, cardiac toxicity (rare fatal)
Immunotherapy	Anti–PD-1 (Nivolumab, Pembrolizumab)	Blocks PD-1 receptor, restores T-cell activity	Metastatic or unresectable melanoma; adjuvant setting	PD-L1 expression (optional), TMB, IFN-γ signature	With CTLA-4 inhibitors, targeted therapy, or chemo	Loss of MHC expression, JAK/STAT mutations, T cell exhaustion	Immune-related AEs: colitis, hepatitis, pneumonitis (can be severe/fatal)
	Anti–CTLA-4 (Ipilimumab)	Blocks CTLA-4, enhances T-cell priming and activation	Metastatic or refractory melanoma	None specific	With PD-1 inhibitors (dual checkpoint blockade)	Severe immune evasion, Treg expansion	Colitis, dermatitis, hypophysitis, enteritis (severe/fatal in rare cases)
Oncolytic Therapy	Talimogene laherparepvec (T-VEC)	Modified HSV-1 virus that replicates in tumors and expresses GM-CSF	Unresectable stage IIIB–IV melanoma (locoregional)	HSV-seronegative status (relative)	With PD-1 inhibitors (under investigation)	Immunosuppressive tumor microenvironment	Flu-like symptoms, injection site pain (generally mild; rare systemic viral spread possible)
Chemotherapy	Dacarbazine, Temozolomide	Alkylating agents causing DNA damage	Previously standard in advanced melanoma	None required	Rarely used; now replaced by immunotherapy/targeted agents	High toxicity, low response rates	Myelosuppression, nausea, fatigue (potentially severe infections)
Radiotherapy	Stereotactic, whole-brain, adjuvant RT	DNA damage, p53-dependent apoptosis	Brain metastases, palliation	None required	With checkpoint inhibitors (for synergy)	Radioresistance (via DNA repair enzymes)	Cognitive impairment, fatigue, dermatitis (rare severe necrosis)
Surgery	Wide local excision, lymph node dissection	Curative in early-stage melanoma	Stage I–II and some stage III	Tumor thickness, ulceration, SLNB results	Adjuvant immunotherapy or radiotherapy in high-risk cases	N/A	Wound complications, lymphedema, infection
Adjuvant/Neoadjuvant	Immunotherapy or targeted therapy	Same as above—used to reduce recurrence risk or downstage tumors	Stage III–IV resectable or high-risk patients	BRAF status, LDH, PD-L1 (optional)	Nivolumab, Dabrafenib/Trametinib as standard options	Similar to primary therapies	Dependent on regimen used (includes severe immune or cardiac events for targeted/immunotherapy)

PD-1/PD-L1 therapies are now first-line in most metastatic or unresectable melanomas. BRAF/MEK inhibitors are preferred for rapid tumor burden reduction in BRAF-mutated patients. Combination immunotherapy (PD-1 + CTLA-4) improves response but increases immune toxicity. T-VEC is limited to superficial lesions and is not systemic. Chemotherapy is largely obsolete unless other options fail or are contraindicated.

**Table 3:** RAF isoforms and mutations with associated cancer types and clinical relevance types according to [90]. All abbreviations employed are defined in the text in the Abbreviations section.

RAF Isoform/Mutation	Cancer Type(s)	Clinical Relevance
B-RAF V600E	Melanoma, Colorectal, Thyroid (Papillary), Ovarian, NSCLC	Highly oncogenic; Targetable with B-RAF and MEK inhibitors; In colorectal cancer, associated with poor prognosis and resistance to B-RAF inhibitor monotherapy
B-RAF Non-V600 Mutations (general)	Melanoma	Targetable with B-RAF and MEK inhibitors
B-RAF F595L	Melanoma, Colorectal, Thyroid (Papillary), NSCLC	Weak/intermediate kinase activity; sometimes respond better to MEK inhibitors rather than B-RAF inhibitors
B-RAF L597Q	Melanoma, Colorectal, Thyroid (Papillary), NSCLC	Weak/intermediate kinase activity; sometimes respond better to MEK inhibitors rather than B-RAF inhibitors
B-RAF G469A	Melanoma, Colorectal, Thyroid (Papillary), NSCLC	Weak/intermediate kinase activity; sometimes respond better to MEK inhibitors rather than B-RAF inhibitors
B-RAF V600K	Hairy Cell Leukemia	Highly sensitive to B-RAF inhibition
C-RAF F133L	Melanoma, Colorectal	May respond to B-RAF inhibitors
C-RAF S257L	Melanoma, Colorectal	May respond to B-RAF inhibitors
A-RAF Rare Mutations	Colorectal, Ovarian, Lung, Pancreatic, Gliomas	Limited therapeutic targeting due to complexity of RAF dimerization; limited treatment options

**Table 4:** Comparison of vemurafenib, trametinib, and imatinib according to [12,132,196,246]. All abbreviations employed are defined in the text in the Abbreviations section.

Feature	Vemurafenib	Trametinib	Imatinib
Drug Class	BRAF inhibitor (ATP-competitive)	MEK1/2 inhibitor (non-ATP competitive)	Tyrosine kinase inhibitor (TKI)
Primary Molecular Target(s)	Mutant BRAF V600E	MEK1 and MEK2 (MAP2K1/2) downstream of BRAF	BCR-ABL (CML, Ph+ ALL) KIT (GIST) PDGFRα/β (DFSP, HES)
Primary Signaling Pathway	MAPK/ERK pathway (RAS–RAF–MEK–ERK)	MAPK/ERK pathway (inhibits downstream of RAF, at MEK level)	BCR-ABL STAT/JAK KIT/PDGFR PI3K/AKT/mTOR MAPK/ERK
Upstream Activators	RAS– Receptor tyrosine kinases (RTKs)	BRAF (WT or mutant) RAS mutations RTKs	BCR-ABL fusion gene SCF for KIT PDGF for PDGFR
Direct Action	Inhibits aberrant BRAF V600E kinase activity to block downstream MEK/ERK signaling	Inhibits MEK1/2 phosphorylation to prevent ERK activation	Inhibits phosphorylation of BCR-ABL, KIT, and PDGFR, blocking their downstream signal transduction
Downstream Effects	↓ MEK1/2 and ERK1/2 phosphorylation ↓ cyclin D1 ↓ proliferation ↑ apoptosis	↓ ERK phosphorylation ↓ proliferation ↑ apoptosis	↓ PI3K/AKT, MAPK, and JAK/STAT signaling ↓ proliferation ↑ apoptosis
Cellular Consequences	G1-phase arrest Decreased cell survival and angiogenesis	G1-phase arrest Reduced tumor growth	Suppression of malignant hematopoietic cell growth Long-term disease control in CML and GIST
Resistance Mechanisms	BRAF amplification NRAS mutation MAPK pathway reactivation PI3K activation	MEK amplification ERK mutations Bypass via PI3K/AKT, RTKs	BCR-ABL mutations (e.g., T315I) Overexpression KIT exon 17 mutations Activation of bypass kinases
Combination Strategies	With MEK inhibitors (e.g., trametinib) With PI3K/mTOR inhibitors Autophagy inhibitors (HCQ)	With BRAF inhibitors With CDK4/6 inhibitors Immunotherapy	2nd/3rd gen TKIs (dasatinib, ponatinib) Allosteric BCR-ABL inhibitors (asciminib) Epigenetic agents
Clinical Biomarkers	BRAF V600E mutation	BRAF V600E/K, NRAS (trial/experimental)	BCR-ABL, KIT, PDGFRA mutations (PCR, FISH, NGS)
Median OS (months)	13.6 (monotherapy); up to 25 (with MEK inhibitor)	~11 (monotherapy); up to 25 (combo)	~12
Median PFS (months)	5.3 (monotherapy); ~12 (combo)	4–5 (mono); 11–12 (combo)	3.5–4
ORR (%)	50–60 (mono); ~70 (combo)	20–25 (mono); 60–70 (combo)	30–50

**Table 5:** Adverse effects of vemurafenib, trametinib, and imatinib with frequency and severity [38,39,40,41,63], where—frequency categories: Very common (>30%), Common (10–30%), Uncommon (1–10%), Rare (<1%);—severity grading: Mild—manageable without dose change; Moderate—may require dose adjustment/supportive therapy; Severe/Fatal—life-threatening, may require therapy discontinuation. All abbreviations employed are defined in the text in the Abbreviations section.

Adverse Effect	Vemurafenib	Trametinib	Imatinib
Arthralgia	Very common (>30%)—Mild–Moderate	–	–
Rash	Very common (>30%)—Mild–Moderate	Very common (>30%)—Mild–Moderate	Common (10–30%)—Mild–Moderate
Photosensitivity	Very common (>30%)—Mild–Moderate	–	–
Fatigue	Common (10–30%)—Mild–Moderate	Very common (>30%)—Mild–Moderate	Common (10–30%)—Mild–Moderate
Diarrhea	–	Very common (>30%)—Mild–Moderate	Common (10–30%)—Mild–Moderate
Nausea	Common (10–30%)—Mild	Common (10–30%)—Mild	Very common (>30%)—Mild
Vomiting	–	–	Common (10–30%)—Mild–Moderate
Pruritus	Common (10–30%)—Mild–Moderate	–	–
Alopecia	Common (10–30%)—Mild	–	–
Edema/Periorbital edema	–	Common (10–30%)—Mild–Moderate	Very common (>30%)—Mild–Moderate
Hypertension	–	Common (10–30%)—Moderate–Severe	–
Cutaneous squamous cell carcinoma/Keratoacanthoma	Common (10–30%)—Severe	–	–
Cardiotoxicity (heart failure, QT prolongation)	Uncommon (1–10%)—Severe/Potentially fatal	Uncommon (1–10%)—Severe	Rare (<1%)—Severe/Potentially fatal
QT prolongation	Uncommon (1–10%)—Severe/Potentially fatal	–	Rare (<1%)—Severe/Potentially fatal
Hepatotoxicity	Uncommon (1–10%)—Severe	–	Uncommon (1–10%)—Severe/Potentially fatal
Cardiomyopathy/decreased ejection fraction	–	Uncommon (1–10%)—Severe	Rare (<1%)—Severe/Potentially fatal
Retinopathy/Retinal vein occlusion	–	Uncommon (1–10%)—Severe/Vision-threatening	–
Interstitial lung disease/Pneumonitis	–	Rare (<1%)—Severe/Potentially fatal	–
Myelosuppression (neutropenia, thrombocytopenia)	–	–	Common (10–30%)—Severe
Muscle cramps	–	–	Common (10–30%)—Mild–Moderate

**Table 6:** Novel melanoma therapies categorized by mode of action and strategy according to [53]. All abbreviations employed are defined in the text in the Abbreviations section.

Mechanism of Action	Treatment Approach
Identifies predictive markers (PD-L1, TMB, IFN-γ, LAG-3, TILs, HLA types, gut microbiota) to personalize therapy and improve treatment outcomes.	Biomarker Discovery and Validation
Employs genetically engineered T cells (e.g., TYP1-targeted) to recognize and kill melanoma cells; currently in preclinical and early clinical stages.	CAR-T Cell Therapy Targeting Melanoma Antigens
Enhances antitumor responses by integrating ICIs with radiotherapy, oncolytic viruses, vaccines, or TILs to overcome immune resistance mechanisms.	Combination Immunotherapy Approaches
Androgen receptor inhibition may boost NK cell activity and improve ICI effectiveness; currently under preclinical evaluation.	Natural Killer (NK) Cell-Based Immunotherapy
Suppresses melanoma cell growth by inhibiting mutations in key driver genes (BRAF, KIT, NRAS, TERT, CCND1); TERT and CCND1 are common in acral melanoma.	Targeted Inhibition of Oncogenic Signaling
Utilizes expanded TILs (e.g., lifileucel) to directly attack tumor cells; effective after ICI and BRAF/MEK inhibitor failure, though limited by cost and toxicity.	Tumor-Infiltrating Lymphocyte (TIL) Therapy

## Data Availability

Data sharing is not applicable to this article.

## Abbreviations

The following abbreviations are used in this manuscript:

ADP	Adenosine Diphosphate
AEs	Adverse Events
AKT	Protein Kinase B
ALL	Acute Lymphoblastic Leukemia
AR	Androgen Receptor
ATP	Adenosine Triphosphate
A-RAF	A-Raf Proto-Oncogene, Serine/Threonine Kinase
B-RAF	B-Raf Proto-Oncogene, Serine/Threonine Kinase
BCR-ABL	Breakpoint Cluster Region-Abelson Fusion Gene
BRAF	v-Raf Murine Sarcoma Viral Oncogene Homolog B1
C-RAF	C-Raf Proto-Oncogene, Serine/Threonine Kinase
CAR-T	Chimeric Antigen Receptor T-cell
CCND1	Cyclin D1
CDK4/6	Cyclin-Dependent Kinases 4 and 6
CML	Chronic Myeloid Leukemia
CTLA-4	Cytotoxic T-Lymphocyte–Associated Protein 4
DFSP	Dermatofibrosarcoma Protuberans
ECG	Electrocardiogram
ERK	Extracellular Signal-Regulated Kinase
FISH	Fluorescence In Situ Hybridization
GAP	GTPase-Activating Protein
GDP	Guanosine Diphosphate
GEF	Guanine Nucleotide Exchange Factor
GIST	Gastrointestinal Stromal Tumor
GM-CSF	Granulocyte-Macrophage Colony-Stimulating Factor
GTP	Guanosine Triphosphate
HCQ	Hydroxychloroquine
HES	Hypereosinophilic Syndrome
HLA	Human Leukocyte Antigen
HSV-1	Herpes Simplex Virus Type 1
ICI	Immune Checkpoint Inhibitor
IFN-γ	Interferon Gamma
ILD	Interstitial lung disease
JAK	Janus Kinase
JAK/STAT	Janus Kinase/Signal Transducer and Activator of Transcription
KIT	Stem Cell Factor Receptor/Receptor Tyrosine Kinase
LAG-3	Lymphocyte-Activation Gene 3
LDH	Lactate Dehydrogenase
MAP2K1/2	Mitogen-Activated Protein Kinase Kinase 1 and 2
MAPK	Mitogen-Activated Protein Kinase
MEK	Mitogen-Activated Protein Kinase Kinase
MHC	Major Histocompatibility Complex
mTOR	Mechanistic Target of Rapamycin
NGS	Next-Generation Sequencing
NK cell	Natural Killer Cell
NRAS	Neuroblastoma RAS Viral Oncogene Homolog
ORR	Objective response rate
OS	Overall survival
P	Phosphate
PCR	Polymerase Chain Reaction
PD-1	Programmed Cell Death Protein 1
PDGF	Platelet-Derived Growth Factor
PDGFRA	Platelet-Derived Growth Factor Receptor Alpha
PDGFR	Platelet-Derived Growth Factor Receptor
PD-L1	Programmed Death-Ligand 1
PFS	Progression-free survival
Ph+	Philadelphia Chromosome–Positive
PI3K	Phosphoinositide 3-Kinase
QT	QT Interval
RAF	Rapidly Accelerated Fibrosarcoma
RAS	Rat Sarcoma Virus Oncogene
RT	Radiotherapy
RTK	Receptor Tyrosine Kinase
SCC	Squamous cell carcinoma.
SCF	Stem Cell Factor
SLNB	Sentinel Lymph Node Biopsy
STAT	Signal Transducer and Activator of Transcription
TERT	Telomerase Reverse Transcriptase
TIL	Tumor-Infiltrating Lymphocyte
TKI	Tyrosine Kinase Inhibitor
TMB	Tumor Mutational Burden
Treg	Regulatory T Cell
TYP1	Tyrosinase-Related Protein 1
V600E/K	Valine to Glutamic Acid/Lysine Substitution at Position 600
WT	Wild Type
